# The cycle ergometer test is not a reliable alternative to the countermovement jump in the assessment of power output

**DOI:** 10.17159/2078-516X/2022/v34i1a12869

**Published:** 2022-01-01

**Authors:** KR Peyper, B Olivier, A Green

**Affiliations:** 1 Department of Sport and Movement Studies, Faculty of Health Sciences, University of Johannesburg, South Africa; 2 Wits Cricket Research Hub for Science, Medicine and Rehabilitation, Faculty of Health Sciences, University of the Witwatersrand, Johannesburg, South Africa

**Keywords:** power, neuromuscular function, rugby union

## Abstract

**Background:**

Rugby union is a physically demanding collision sport that requires optimal neuromuscular function for maximal power output, with mechanical power an integral component of performance. Peak power (P_p_) and relative P_p_ are parameters of neuromuscular function commonly assessed through the countermovement jump (CMJ) as a measure of fatigue. The Wattbike cycle ergometer test (CET) is a non-load bearing method of evaluating lower limb power. The cost-effective CET could therefore offer a viable alternative to the CMJ.

**Objectives:**

This study aimed to determine the concurrent validity of the CMJ and CET.

**Methods:**

Thirty-eight professional rugby union players performed twelve CMJs on a force platform with four loads (bodyweight: BW-CMJ; 20kg: 20-CMJ; 40kg: 40-CMJ and 60kg: 60-CMJ) and a six second peak power (6PPO) CET assessment on a Wattbike ergometer.

**Results:**

CMJ power outputs were [BW-CMJ: P_p_ − 3101±648 W; 20-CMJ: P_p_ − 2724±513 W; 40-CMJ: P_p_ − 2490±496 W; 60-CMJ: P_p_ − 2238±366 W] and CET [P_p_ – 1310±161 W]. None of the CMJ-P_p_ values showed relationships with any CET power variables. Large (r = 0.51–0.63; p = 0.000 – 0.001) relationships were found to be between relative CMJ and relative CET power outputs. Bland-Altman plots, which were used to determine the level of agreement between the two assessments, showed the agreement between the tests was poor.

**Conclusion:**

Though positive relationships existed between relative CMJ and relative CET power variables, analyses of the level of agreement in the Bland-Altman plots suggest that the two power assessment methods are not interchangeable measures of power.

Rugby union is an intermittent, power-based contact sport involving high-impact collisions between opposing players, repeated high-intensity bouts and continuous ballistic movements. ^[1]^ With condensed schedules, professional rugby union players and coaches are continually looking for ways to improve performance and prevent injury by improving fundamental physical qualities such as strength and power.^[2]^

Neuromuscular function is commonly used to measure the manifestation of fatigue in elite athletes.^[2]^ Fatigue can be described as an exercise-induced deterioration in performance ^[2]^, with studies showing that both acute ^[3]^ and chronic ^[4]^ workloads can affect neuromuscular function. Mechanical power, a function of neuromuscular performance, is an integral component in the movements, collisions and success of rugby union athletes. The countermovement jump (CMJ) and Wattbike six-second peak power output (6PPO) tests are effective assessments in the measurement of mechanical power and changes in neuromuscular function, which can be measured with relative simplicity and at minimal additional fatigue to the athlete.

The assessment of mechanical power helps to quantify the ability of the athlete to execute athletic movements.^[5]^ The force plate has become the gold standard in the measurement of lower limb power due to its ability to measure different ground reaction forces and related metrics.^[6]^ However, movements such as the CMJ are ballistic in nature with a large eccentric component and emphasis on the stretch-shortening cycle. In the days following match play or training, the eccentric nature of the CMJ becomes less favourable in athletes suffering from severe delayed onset of muscle soreness (DOMS) due to discomfort and a potential increase in the risk of injury.

The Wattbike cycle ergometer has recently been suggested as a non-load bearing method to evaluate lower limb power.^[7,8]^ Its predominantly concentric and less ballistic mechanism, could prove to be a replacement for the CMJ as a measure of neuromuscular function and fatigue. Commonly used cycle ergometer tests (CET) are the Wingate thirty-second anaerobic test, Wattbike thirty-second anaerobic power and Wattbike 6PPO tests.^[9]^ Previous literature shows athletes produce maximum power within the first six seconds of a CET ^[7]^, making the Wattbike 6PPO test an effective test of maximal power without the fatiguing effects of the thirty-second anaerobic tests .^[9]^ Moreover, positive relationships between the CMJ and CET power outputs have been reported. ^[3,8]^

However, there is limited literature on the relationship between force plate CMJ variables and CET outputs, and the benefits which such relationships may exhibit. The investigation on whether relationships exist between the F-v profiles of the athletes and the CET variables could also provide insight into whether the athletes are performing optimally. Therefore, the aim of the study was to determine the concurrent validity of the CMJ and Wattbike CET power evaluations.

## Methods

### Participants

Healthy professional male rugby union players were invited to participate in the study. The study was approved by the institutional ethics committee (REC 01-55-2019). Participants were injury-free at the time of testing and provided written informed consent prior to testing.

### Testing procedures

Data collection was carried out at a professional rugby union training facility in Johannesburg, South Africa. Participants attended two different testing sessions in a randomised order on separate days during the off-season. Test days were performed no more than three days apart, with the order of test days randomised. In both testing procedures, participants performed a standardised 10-minute investigator led warm-up consisting of submaximal cycling, dynamic stretching and a series of submaximal countermovement jumps.

### Countermovement jump (CMJ)

Participants performed a modified CMJ protocol consisting of three CMJs at each random added load of 0, 20, 40 and 60 kg respectively, with a rest time of three minutes between jumps. Varying loads were utilised to determine where the strongest relationship may lie between the CMJ and CET. A total of 12 CMJs were performed. An additional load was added to a barbell across the shoulders, while for the 0 kg jumps a plastic pipe (<1kg) was utilised. Participants were instructed to use a self-selected depth in the eccentric phase of the CMJ after which the concentric phase was performed as quickly as possible, keeping the legs fully extended in the air. Any jumps which were inaccurately performed were reattempted after no more than five minutes of rest. All successful CMJs were recorded at 1000 Hz using a force plate (Bertec Type 4060-05, Bertec Corporation, Columbus, OH, USA). The highest attempt at peak force production of the three for each load was utilised for the final analysis.

### Cycle ergometer test (CET)

The six-second CET test procedure involved two six-second maximal sprints on a Wattbike Pro (Wattbike Ltd, Nottingham, UK) with saddle and handlebars as well as air and magnetic resistance individually set in accordance with manufacturers’ guidelines.^[8]^ The second test followed a rest of no more than five minutes and no verbal encouragement was given for either attempt. The best score of the two sprints was used for the final analysis.

### Data analyses

All jump analyses were based on the participants’ best CMJ performance at each load. From these jumps, the following variables were obtained: peak force (F_p_), jump height (JH) and peak velocity (V_p_). F_p_ was the highest force produced in the concentric phase of the CMJ. Power was calculated at each time point on the graph and peak power (P_p_) was the maximum value during the concentric phase. CMJ mean power was calculated as the average instantaneous power over the concentric push-off phase of the jump. Peak power, mean power (P_m_), relative P_p_ and relative P_m_ outputs from the CET were captured from the ergometers’ onboard computer. Finally, theoretical maximal force (F_0_), theoretical maximal velocities (V_0_), theoretical peak power output (P_o_) and gradient (S_fv_) were calculated for each participant’s F-v profile utilising the mean power data calculated from the force plate data.

### Formulae

V_p_ - (calculated utilising the trapezoid rule: $\int_{ti}^{tf}\frac{GRFz-mg}{m}dt$^[14]^, where *GRF* = ground reaction force (N), *m* = mass (kg), *g* = gravitational acceleration (m.s^−2^), *ti* = initiation time and *tf* = final time). ^[11]^


$$\begin{array}{c} {\text{P}}_{\text{p}}=({\text{F}}_{\text{p}}\times {\text{V}}_{\text{p}}).\\ {\text{P}}_{\text{o}}=(\text{calculated as}\frac{\text{F}0.\text{v}0}{4}). \end{array}{}^{\left[12\right]}$$

Bland Altman plots were used to describe the limits of agreement between relative P_p_ and relative P_m_ as measured in CMJ and on the Wattbike. The bias in the plots was determined as the average difference between the two assessments, while the upper and lower limits of agreement were set at 95%. The plots were utilised to evaluate bias between the two measurements and highlight that agreement is more a question of estimation, and not a form of hypothesis testing.^[11]^ Microsoft Excel was used to compile the Bland Altman plots.

### Statistical analysis

Data distribution was determined utilising a Shapiro-Wilk test. All data are presented as mean ± standard deviation. Pearson’s and Spearman’s correlations were used to determine the relationships between force plate metrics and power outputs on the Wattbike. All data were analysed using Statistical Package for Social Science software (SPSS, IBM Version 25.0. Armonk, NY: IBM Corp). Qualitative descriptors were represented as trivial (<0.1), small (0.1–0.3), moderate (0.3–0.5), large (0.5–0.7), very large (0.7–0.9).

## Results

A sample of thirty-eight healthy professional male rugby union players from the greater Johannesburg area (age: 20.2±1.6 years, stature: 183±7 cm, mass: 95.5±13.1 kg) participated in this study. The average CMJ-F_p_ values increased as the load was increased due to the added load and the acceleration of gravity. However, due to the added load and increased mass of the system CMJ-V_p_, CMJ-P_p_, and CMJ-JH all decreased as the system became more difficult for the participants to move (Table 1).

The Wattbike CET variables were calculated as CET-P_p_: 1310±161 W; CET-P_m_: 1160±155 W; relative CET-P_p_: 13.74±1.71 W.kg^−1^ and relative CET-P_m_: 12.16±1.62 W.kg^−1^.

Correlations were calculated between the outputs determined in the CMJ and CET tests (Table 2). Large positive relationships (r = 0.52–0.66) were found between F_p_ in all the CMJs and CET-P_p_ and CET-P_m_. Positive, moderate to large relationships (r = 0.34–0.68) were found between CMJ-V_p_ values and relative CET-P_p_ and CET-P_m_ values. The CMJ-P_p_ values exhibited no significant relationships with any of the CET variables; all correlation data are presented in table 2. Large (r = 0.51–0.63) relationships were found between relative bodyweight CMJ-P_p_ and relative CET-P_p_ and CET-P_m_ variables, large (r = 0.51–0.63) relationships were also found between relative CMJ-P_m_ and relative CET-P_p_ and CET-P_m_. Additionally, only moderate relationships (r = 0.32–0.44) were found between CMJ-JH in the loaded jumps and CET-P_p_ and CET-P_m_ (Table 2).

**Fig. 1:**
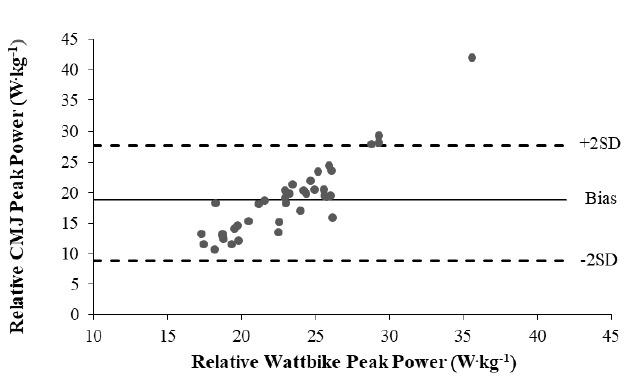
Bland-Altman plot indicating the heteroscedastic distribution of data points between the limits of agreement between relative peak power values in the countermovement jump (CMJ) and cycle ergometer test (CET) data in 38 professional rugby union players.

**Fig. 2:**
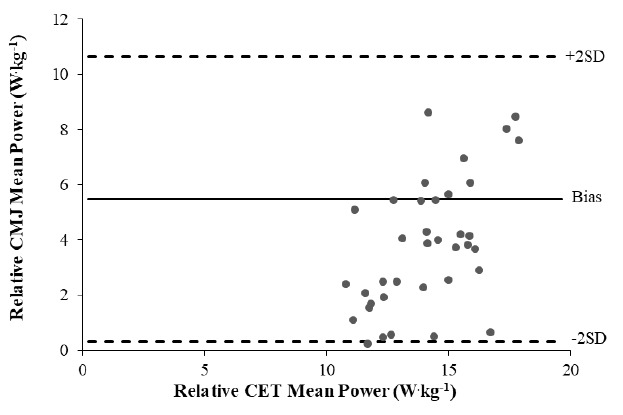
Bland-Altman plot indicating the equal scattered distribution of data points above and below the bias, between relative mean power values in the countermovement jump (CMJ) and cycle ergometer test (CET) data in 38 professional rugby union players.

Bland-Altman plots were used to determine the level of agreement between relative peak power (Figure 1) and relative mean power (Figure 2) values in the CMJ and CET. The limits of agreement (LOA) in the relative P_p_ plot were 8.8 W.kg^−1^ and 27.7 W.kg^−1^ for the lower and upper limits, respectively, with a bias of 18.3 W.kg^−1^. In relative peak power (Figure 1), the Majority of the points have a heteroscedastic appearance, indicating the increase in error was directly proportional to the increase in force. A single outlier and three points are noted outside of the upper LOA. The LOA in the relative P_m_ plot were 0.3 W.kg^−1^, and 10.6 W.kg^−1^ for the lower and upper limits, respectively, with a bias of 5.5 W.kg^−1^. The P_m_ plot exhibited a less uniform pattern, with two outliers and two values lying above the upper LOA (Figure 2).

The CET power variables were compared to P_0_ (34.8±4.9 W.kg^−1^) and S_fv_ (gradient: −11.7±4.3 N.s.m^−1^.kg^−1^) calculated from the force-velocity profiles. Only the CET relative peak power (r = 0.43) and relative mean power (r = 0.37) indicated moderate relationships with P_o_ (p < 0.05). Neither CET peak (r = −0.05) or mean (r = −0.13) power exhibited any significant relationship with P_o_ or S_fv_ (p > 0.05).

## Discussion

The purpose of this study was to examine the concurrent validity of the CMJ and CET in professional rugby union players. Numerous positive, moderate to large relationships were found between the CMJ and CET variables. However, no significant relationships were found between CET- P_p_, CET-P_m_, CMJ-RFD or bodyweight CMJ-JH and the CET power outputs. Bland-Altman plots showed little agreement between the relative CMJ and relative CET power variables.

Rugby union is a game that requires multiple physical performance traits to initiate, evade and dominate various collision moments. Measurement of force, velocity and power variables helps to quantify the ability of athletes to perform such tasks. Owing to varying ranges and methods of jump loads used across studies ^[11,13]^, it was difficult to draw comparisons with the present study’s jump variables. The average F_p_ results in the bodyweight CMJs were lower than those found in previous literature on elite rugby union players.^[14]^ The average V_p_ results were similar to results previously obtained in studies conducted on rugby players.^[5,15]^ However, the average P_p_ results were not supported by previous literature on professional rugby union players.^[14,16]^ These findings could be due to the participants in the present study’s lower F_p_ production than in the previously mentioned research, which could have been caused by a number of factors. The younger average age in the current sample could indicate that the athletes had not reached full maturity in strength training. Previous research indicated similar findings of lower F_p_ output in young rugby union professionals. ^[17]^ The training methods and game plan utilised by the athletes and organisations in question may differ from other athletes and organisations. The average relative P_p_ results of the present study also indicated considerably lower results than previous literature ^[15]^, however, this was expected due to the lower F_p_ production of the athletes in the study. When comparing the peak power and relative power outputs, it is important to note that relative power could provide a more accurate comparison of performance, especially in rugby union where performance variables and anthropometry differ greatly between positions.^[1]^

Jump height is a standard measurement in most power-based sports, with some sports placing an emphasis on jump height when selecting players. The average bodyweight CMJ-JH in the present study exhibited similar results to those found in a previous study on rugby league players.^[16]^ However, measuring CMJ-JH alone may not provide sufficient data when assessing performance in athletes as previous research found that power and rate of force development (RFD) were more related to sport-specific movements and could provide a more accurate indication of the athletes’ ability to produce powerful sport-specific movements.^[18]^ The RFD results, as with the P_p_ results, in the present study were, however, also found to be considerably lower than those in previous research on professional rugby union players.^[14]^ As previously mentioned, the lower force production by the athletes in the present study could have affected jump variables such as RFD.

Force-velocity profiles were established using CMJ data in order to determine whether any relationship existed between CET power variables and the F-v profiles. The absence of any relationship between the theoretical peak power in F-v profiles and CET power outputs suggested that the participants in the study may not have exhibited optimised F-v profiles. These findings are in line with the previous argument that the lower force production of the athletes in this study could affect the jump variables, and subsequently the F-v profiles. Therefore, further investigation is needed on CET and F-v profiles.

The Wattbike cycle ergometer is amongst the industry’s leading devices for assessment of lowerlimb power. The results for the present study in the CETs were supported by previous literature on professional rugby union players.^[19]^ The CMJ and CET differ in mechanism, with the CMJ being more ballistic and bilateral in nature than the CET. This difference in mechanism is indicated by the vast difference in peak- and mean power output figures between the assesments. However, the CMJ and CET are both accurate tests of lower limb power output and it was therefore important to determine whether any relationship existed between the two assessment methods.

The CMJ is the most frequently used measurement of neuromuscular function.^[4,13]^ Previous literature on rugby union players ^[8]^ and Australian rules football players^[3]^ have all reported positive relationships between CMJ and CET power outputs. Though no relationship existed between CMJ and CET peak and mean power outputs in the present study, the large relationships found between the relative power outputs show that the CET may be a suitable alternative to the CMJ when assessing power outputs. However, the CET is limited by the number of variables it can assess. While the CMJ can assess eccentric and concentric phases of motion and components of the stretch-shortening cycle, the CET can only assess the concentric phase. Bland-Altman plots were therefore used to determine the level of agreement between the relative P_p_ outputs and relative P_m_ outputs. Though the CET presents a more viable and cost-effective alternative to the force plate CMJ, the insufficient agreement shown between the two assessment methods indicates that the two assessments are not interchangeable as measures of lower limb power. Specifically, the large biases and wide limits of agreement indicate that, although the assessments can both be utilised to measure lower limb power, they should not be used interchangeably.

### Practical applications

The CMJ and CET procedures utilised in the present study are both independent accurate measures of muscular power development as they assess lower limb power utilising different mechanisms. The CET could be considered a viable, non-interchangeable alternative to the force plate CMJ. Therefore, coaches and trainers are advised to adhere to single modes of muscular power testing.

## Conclusion

The present study sought to compare the CMJ variables and CET power outputs in professional rugby union players. Only the relative CET power metrics shared large relationships with relative CMJ metrics. However, the lack of agreement between the two tests indicates that the Wattbike 6PPO test should not be used as an interchangeable alternative power assessment.

## Figures and Tables

**Table 1:** Force, velocity, power, rate of force development and jump heights for weighted countermovement jumps

	Bodyweight	20kg load	40kg load	60kg load
**Peak force (N)**	2389 ± 327	2537 ± 381	2665 ± 314	2863 ± 322
**Peak velocity (m.s** ^**−1**^ **)**	2.87 ± 0.37	2.53 ± 0.20	2.30 ± 0.22	2.06 ± 0.15
**Peak power (W)**	3101 ± 648	2724 ± 513	2490 ± 496	2238 ± 366
**Relative peak power (W.kg** ^**−1**^ **)**	32.63 ± 6.80			
**Relative mean power (W.kg** ^**−1**^ **)**	18.20 ± 3.87			
**Rate of force development (N.s** ^**−1**^ **)**	5951 ± 3951	5286 ± 3620	3971 ± 2754	3533 ± 2700
**Jump height (m)**	0.35 ± 0.06	0.29 ± 0.05	0.23 ± 0.05	0.21 ± 0.20

**Table 2:** Correlations between countermovement jumps (CMJ) and cycle ergometer test (CET) data

CMJ parameters	CET			

	P_p_	P_m_	Relative P_p_	Relative P_m_
**F**_**p**_ **BW**	0.515**	0.554**	−0.029	0.055
*p-value*	*0.001*	*<0.001*		
**F**_**p**_ **20kg**	0.522**	0.604**	−0.011	0.075
*p-value*	*<0.001*	*<0.001*		
**F**_**p**_ **40kg**	0.627**	0.655**	−0.025	0.054
*p-value*	*<0.001*	*<0.001*		
**F**_**p**_ **60kg** #	0.562**	0.577**	−0.101	−0.022
*p-value*	*<0.001*	*<0.001*		

**V**_**p**_ **BW** #	0.184	0.067	0.680**	0.550**
*p-value*			*<0.001*	*<0.001*
**V**_**p**_ **20kg**	0.041	0.048	0.438**	0.405*
*p-value*			*0.006*	*0.01*
**V**_**p**_ **40kg** #	0.122	0.098	0.339*	0.412*
*p-value*			*0.04*	*0.01*
**V**_**p**_ **60kg** #	0.090	0.082	0.535**	0.563**
*p-value*			*0.001*	*<0.001*

**P**_**p**_ **BW** #	0.168	0.135	0.251	0.206
**P**_**p**_ **20kg**	0.089	0.090	0.138	0.188
**P**_**p**_ **40kg**	0.070	0.045	0.172	0.251
**P**_**p**_ **60kg**	0.100	0.072	0.200	0.264

**Relative P**_**p**_ **BW** #	0.194	0.136	0.631**	0.507**
*p-value*			*<0.001*	*0.001*
**Relative P**_**m**_ **BW**	0.196	0.138	0.634**	0.510**
*p-value*			*<0.001*	*0.001*

**RFD BW** #	−0.203	−0.243	−0.096	−0.055
**RFD 20kg** #	−0.185	−0.174	0.120	0.150
**RFD 40kg** #	−0.236	−0.131	0.142	0.220
**RFD 60kg** #	−0.044	−0.052	0.120	0.170

**JH BW**	0.284	0.297	0.008	0.014
**JH 20kg**	0.346*	0.324*	−0.021	0.080
*p-value*	*0.03*	*0.05*		
**JH 40kg** #	0.385*	0.435**	−0.092	0.012
*p-value*	*0.02*	*0.006*		
**JH 60kg** #	0.340*	0.320	−0.006	0.057
*p-value*	*0.04*			

**S**_**fv**_	0.045	−0.048	−0.114	−0.212
**P**_**o**_ #	−0.053	−0.127	0.431**	0.370*
*p-value*			*0.007*	*0.02*

* indicates p < 0.05;

** indicates p<0.01;

# indicates Spearman’s ranked correlations.

BW, bodyweight; F_p_, peak force; V_p_, peak velocity; P_p_, peak power, P_m_, mean power; RFD, rate of force development; JH, jump height; S_fv_, gradient; P_o_, theoretical peak power output.
